# Linear Regression Model to Identify the Factors Associated with Carbon Stock in Chure Forest of Nepal

**DOI:** 10.1155/2018/1383482

**Published:** 2018-04-03

**Authors:** Ira Sharma, Sampurna Kakchapati

**Affiliations:** ^1^Prince of Songkla University, Pattani Campus, Pattani, Thailand; ^2^Nepal Institute of Health Sciences, Jorpati, Kathmandu, Nepal

## Abstract

Use of woody plants for greenhouse gas mitigation has led to the demand for rapid cost-effective estimation of forest carbon stock and related factors. This study aims to assess the factors associated with carbon stock in Chure forest of Nepal. The data were obtained from Department of Forest Research and Survey (DFRS) of Nepal. A multiple linear regression model and then sum contrasts were used to observe the association between variables such as stem volume, diameter at breast height, altitude, districts, number of trees per plot, and ownership of the forest. 95% confidence interval (CI) plots were drawn for comparing the adjusted carbon stocks with each of the factors and with the overall carbon stock. The linear regression showed a good fit of the model (adjusted *R*^2^ = 83.75%) with the results that the stem volume (sv), diameter at breast height (dbh), and the number of trees per plot showed statistically significant (*p* value ≤ 0.05) positive association with carbon stock. The highest carbon stock was associated with sv more than 199 m^3^/ha, average dbh more than 43.3 cm/plot, and number of trees more than 20/plot, whereas the altitude, geographical location, and ownership had no statistical associations at all. The results can be of use to the government for enhancing carbon stock in Chure that supports both natural resource conservation and United Nations-Reducing Emission from Deforestation and Forest Degradation program to mitigate carbon emission issues.

## 1. Introduction

Forest ecosystems are crucial for global carbon context. A forest is estimated to contain 80% of aboveground and 40% of below ground terrestrial carbon stock [1]. Forest ecosystem stores 20% to 50% more carbon as compared to other ecosystems due to its woody character and long lifespan [2]. The mature forests absorb carbon dioxide (CO_2_) from atmosphere while growing plants store carbon in wood, leaves, and soil. For the tropics, diameter at breast height (dbh) alone explains more than 95% of the variation in aboveground tropical forest carbon stocks, even in highly diverse regions [3].

The loss of forests, primarily in tropical developing countries, accounted for 12 percent of global Green House Gas (GHG) emissions from 2000 to 2005 [4]. Deforestation alone is a cause of 17.4 percent of global carbon gas emission and 3rd largest source of emission in 2004 [5]. Enhancing carbon stock for reducing greenhouse gas effect is today's strategy to mitigate climate change problems. Reducing Emission from Deforestation and Forest Degradation (REDD), a United Nations- (UN-) REDD Collaborative program in developing countries, is of central importance in efforts to combat climate change effects and also provides a clue to sustainable forest management and conservation of carbon. The factors associated with carbon content in forest, therefore, are essential to understanding for keeping a higher carbon stock. Moreover, a sustainable forest resource managed at local and national level is rewarded with economic incentives for carbon sequestration services through REDD+ program and it offers an opportunity for developing countries to generate livelihood benefits for rural poor people, a triple win-win situation [6]. The studies provide evidences that several factors affect the amount of carbon stock of the forest, like stem volume and dbh [7], density of tree [2], altitude [8, 9], canopy [10], species [11], soil [2], and ownership [9, 12]. Carbon content in live wood is essential for quantifying the forest carbon stocks. The wood carbon content varies substantially due to physical factors (temperature, rainfall, land form, altitude, soil, etc.), biological factors (species diversity, wood volume, stem diameter, the number, and density of trees), and social factors (human being and their socioeconomic and cultural affairs). The developing countries need a robust stock of carbon in the forests for successful implementation of climate change mitigation policies of REDD+. There are ample studies to accurately quantify forest carbon at different levels [3, 13, 14]. However, to our knowledge, relatively fewer studies [12, 15] focus on the factors affecting the carbon content in forest. Nepal is an economically weak country and forest is one of the major natural resources of national income and an essential livelihood resource for local people. There is a need to access the factors that significantly associate with the carbon content in forest trees, which would support sustainable management of forest resource and increment of its carbon content.

Forest plays role to control erosion of land resources as soil, sand, and the gravel, especially in Nepal, where the natural drainage from north to south easily erodes tons of those resources every year. Extending from east to west of Nepal, Chure is a semiarid area, covering 12.8% of total land of the country and 73.4% of Chure land is occupied by the forests [16]. The area has deciduous type of vegetation, extending through 36 districts. It consists of unconsolidated sedimentary rocks which make it geologically fragile and also associated with heavy rain, high floods, and landslides. Therefore, government of Nepal has recently declared this belt as Environmental Conservation Area based on Environmental Protection Act 2015 [17]. A need to identify the factors associated with carbon content of forest trees in “Chure” range of Nepal is realized not only for the natural resource conservation but also to support the REDD+ program of UN. Globally, most of the studies concentrate on the carbon content of tropical forests; this study is carried out in a subtropical region. The main objective of the study is to understand the association of carbon content with six different predictors, stem volume (sv), diameter at breast height (dbh), number of trees (tn), ownership, altitude, and the location around Chure region of Nepal by using linear regression model.

## 2. Methodology

### 2.1. Site Description

**Figure 1:**
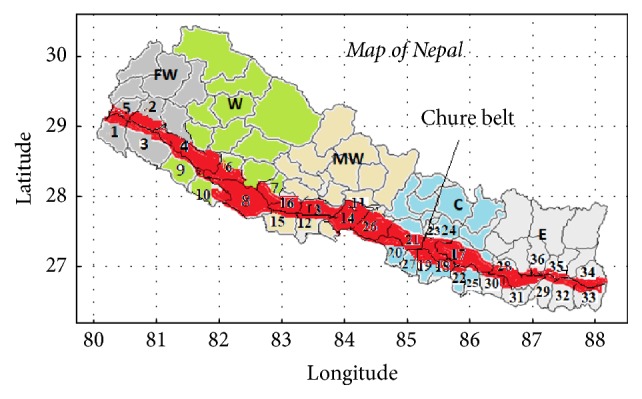
Map of Nepal showing Chure region (red belt) with 36 districts within 5 development regions. FW = far western, W = western, MW = mid-western, C = central, and E = eastern.

This study is carried out in the Chure belt (Figure 1), also known as Shiwalik, of Nepal. It is the youngest mountain range in Himalayas. It lies in North of Terai belt, running entire length of Nepal, from east to west, along the southern flank of Himalayas. The area extends from 80.15°E to 88.18°E and 26.62°N to 29.16°N. It is 20–30 km wide and almost 1000 km long. It extends through 36 districts [18]. The altitude ranges from 92 m to 1955 m and the breadth is 10 km to 50 km. This area is characterized by erodible, fragile, and dry soil [19].

### 2.2. Data Collection and Sampling

**Figure 2:**
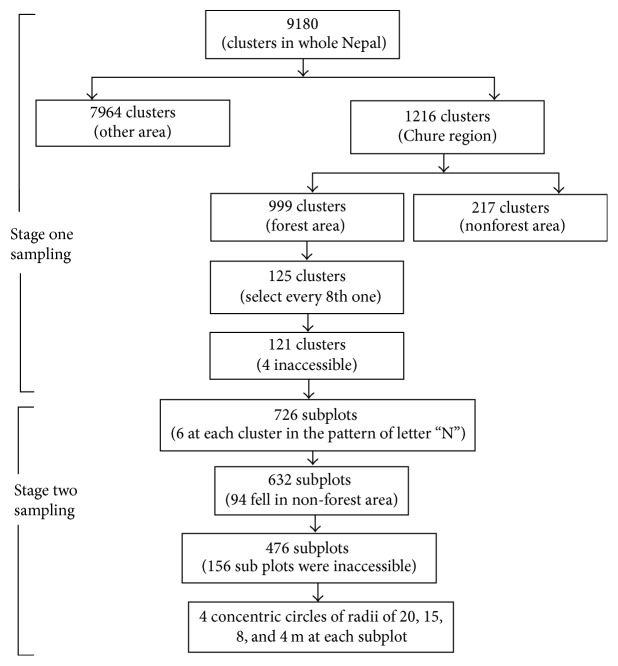
Methodological flow of two-stage sampling.

The data was collected in two-phase systematic sampling method (Figure 2). Firstly, 9180 clusters of 4 × 4 km^2^ grids were made for the entire country. The grids were superimposed on a high resolution (5 × 5 m^2^) Rapid Eye Satellite Image with the help of Google Earth Image and the topographic maps of the country. From each grid knot, 6 sample subplots were projected. Those subplots were initiated from far western part of Nepal. There were 55,358 sample subplots. The subplots lie systematically from south to north (150 m apart) and from west to east (300 m apart). The subplots were numbered 1 to 6, from south to north (northing) and then west to east (easting), in a pattern of letter “N.”

There were 1216 clusters observed in Chure belt. These clusters were divided into two groups: forest and nonforest. If a cluster had at least one subplot with forest cover, the cluster was grouped as forest. Hence, there were 999 clusters of forest and the remaining were nonforest.

In the second phase, the forest clusters were systematically selected choosing every eighth forest cluster, thus resulting in 125 clusters. Among them, four were inaccessible limiting the forest cluster to be 121 (12.1% of total 999 forest clusters), meaning 726 subplots. But 632 subplots were on forest land; of these just 476 were included in this study because 156 were located in either a core protected area or geographically inaccessible region.

### 2.3. Nested Sample Plots

**Figure 3:**
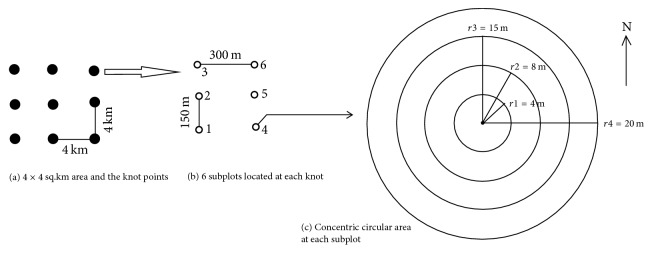
Plots (a), subplots (b), and four concentric circular areas with varying radii (c).

The sample areas selected for the study at 4 × 4 km^2^ plots, subplots, and finally concentric circular area levels are shown in Figures 3(a), 3(b), and 3(c). At each subplot, four concentric circles of radii (*r*) 20 m, 15 m, 8 m, and 4 m were made to enumerate trees with diameter at breast height (dbh), 30+ cm, 20–29.9 cm, 10–19.9 cm, and 5–9.9 cm, respectively. The total area for tree count (within radius 20 m) is 1257 m^2^, which is calculated by

(1) $$\begin{array}{c} A=\frac{{\pi d}^{2}}{4}. \end{array}$$

Here, *A* is the total area of circle with outermost radius *r* which is 20 m at each subplot and *π* is a constant. The diameter (*d*) (in (1)) is calculated as double the *r* value (*d* = 2*r*). For the diameter at breast height (dbh), stem volume, basal area, and the height of the trees in the forest were calculated for each concentric circle with respect to its diameter class and finally averaged to get single value for that particular subplot.

### 2.4. Measurements

#### 2.4.1. Diameter at Breast Height (dbh)

The dbh is measured at a breast height of 1.3 m from the base of the tree, from where the circumference is taken. It is calculated by taking an average dbh (cm) of all trees in every concentric circle of each subplot. Then the diameter is calculated by

(2) $$\begin{array}{c} d=\frac{c}{\pi }. \end{array}$$

Here, *d* is the diameter of tree at breast height, *c* is circumference, and *π* is a constant.

#### 2.4.2. Height

The height models were prepared for tree species and species groups in the Chure. The height is measured in two steps. First, the height of only the sampled trees, at every 5th position, was taken which included all possible species. The samples were systematically chosen at every 5th position tree. To avoid the chance of missing any species, the additional number of trees was selected wherever needed. Hence, in second step, the height of those sampled trees was fitted with nonlinear, mixed effect model, to establish the relationships between the dbh and total heights of trees using the* Lmfor *package in R Software [20]. The best fitted models for all available species were applied to find the height of the trees [16]. For species having too few numbers of sample trees for modeling alone, grouping had been done based on their family, genus, and existing height-diameter observations. This height was then averaged, on species basis in each subplot, and used to calculate per subplot stem volume.

#### 2.4.3. Stem Volume

For stem volume, the average basal area of tree was calculated from, the species specific, stem diameter over the bark at a height of 1.3 m. First, the average diameter per hectare per plot is calculated, which is used to find basal area (in m^2^) of trees per plot, by using formula (1). Finally, this area is used to calculate the volume of the stem by following allometric equation developed by [21]

(3) $$\begin{array}{c} ln\left(\;v\;\right)=a+b\cdot \ln\left(\;d\;\right)+c\cdot \ln\left(\;h\;\right). \end{array}$$

Here, *v* is the tree volume per hectare (m^3^/ha), *h* is the height of the trees (m), and *a* is the average basal area (m^2^) of the trees per hectare per subplot. *a*, *b*, and *c* are the coefficients depending on the species [21].

#### 2.4.4. Biomass

The tree stem biomass is calculated by species specific stem density values of the stem. The stem density is the air dried density in kg/m^3^ [21]. Hence the stem biomass is calculated by the following equation:

(4) $$\begin{array}{c} Stem\;\;biomass\left(\;M\;\right)=\ln\left(\;v\;\right)\times Stem\;\;Density. \end{array}$$

This is the air dried biomass of the stem, which is converted to oven dried one by using the conversion factor 0.91 for this value [22, 23] and a carbon ratio factor 0.47 [24].

#### 2.4.5. Carbon Stock

With the help of total stem volume per subplot (m^3^/ha), carbon content in each subplot is calculated by applying conversion factor of 0.5 [24, 25] and total carbon stocks per plot (tC/ha) is estimated. In fact there were 281 tree species and 177 shrub species enumerated during study.

#### 2.4.6. Altitude

The altitude of the study site was measured in terms of meter (m) by using altimeter.

#### 2.4.7. Ownership

Community forest is government owned and local community managed forest while another forest includes all types of forests, for instance, private, government, religious, and conserved forests. Out of 476 forest subplots, almost 52.31% were community managed forests.

### 2.5. Statistical Analysis

For statistical analysis, the stem volume (m^3^/ha) was grouped into 3 levels based on the preliminary description of data (the first quartile, mean, and the third quartile), that is, <91, 91–199, and >199. The dbh (cm/plot) was categorized on similar principle and three categories were made: <27.1, 27.1–43.4, and >43.4. The number of trees was categorized into two: <20 trees/plot and >20 trees/plot. Similarly, altitude was categorized into three: low (<500), medium (500–1000), and high (>1000). In this study, only 25 districts have been selected and those districts are categorized into 5 groups based on the 5 development regions. The 5 groups are far western (FW) with 3 districts, mid-western (MW) with 5 districts, western (W) with 4 districts, central (C), with 7 districts, and eastern (E) with 6 districts. The last, ownership of the forest, was categorized into two groups: community forest and the others.

The associations between carbon stock in forest and six determinants were performed by multiple linear regression model (additive method). The form of equation for the model is

(5) $$\begin{array}{c} y=\beta_{0}+\beta_{1}x_{1}+\beta_{2}x_{2}+\cdots +\beta_{n}x_{n}+\varepsilon . \end{array}$$

Here, *y* is the outcome, stem carbon stock in the forest, *β*_0_ is the constant, *β*_1_, *β*_2_,…, *β*_*n*_ are the coefficients of the respective predictors *x*_1_, *x*_2_,…, *x*_*n*_, *n* is the number of variables, and *ε* is the error term. Sum contrasts [26] were used to determine 95% confidence intervals (CI) of the variables for comparing the adjusted carbon stock within each factor with overall carbon stock. The CI for factor-specific carbon stock was obtained from the model divided naturally into three groups according to their location, entirely above the mean, around the mean, and entirely below the mean. This trichotomy was used to classify the variable subgroups into high or low association with carbon stock of the forest. All data analysis and graphical displays were carried out using R Statistical Programing [27].

## 3. Results

The study area had total 281 tree species and there were on average 20 trees in each subplot. The average volume of stem is 153.99 m^3^/ha and the average number of stems having dbh of ≥5 cm was 731 per hectare. Out of 476 forest subplots, almost 52% were community owned forests (Table 1).

The total carbon stock in Chure forest was 76.67 tC/ha, excluding the carbon of litter, debris, and the soil component. The maximum total stem volume of live trees was 154.40 m^3^/ha. The number of stems having diameter dbh ≥ 5 cm is 731.41/ha. The average dbh is 36.35 cm. The altitude ranged from 124 m to maximum 1632 m in the study plots with a mean elevation of 513.30 m. The mean frequency of trees per plot is nearly 20 (Table 2).

**Figure 4:**
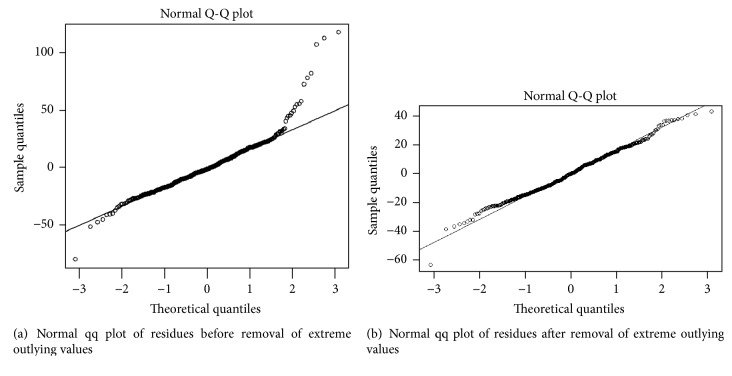


The data were, then, fitted with the multiple linear regression (additive) model. Before analyzing the model results, normality assumption of the residuals is required for checking and, hence, the model fitting was determined by the linearity in the plot of deviance residuals against normal quantiles. Normal quantile-quantile (qq) plot is a probability plot, which is a graphical method for comparing two probability distributions by plotting residuals against their theoretical quantiles.  Figure 4(a) shows the qq plot showing most of the residuals lying on the diagonal line except some values at the upper extreme of the distribution, with the adjusted *R*^2^ 78.6%. To get rid of these extreme outlying values, the residuals above 40 were removed which caused eliminating 15 observations out of 477. Hence only 462 observations were then subjected for multiple linear regression models. Then, the normal qq plot in Figure 4(b) showed that the points have almost followed the diagonal line suggesting the normality of data. The model, then, showed a better fit with the data (adjusted *R*^2^ = 83.8%) having six predictors and outcome variables. The detail of variable parameters from model is shown in Table 3.

**Figure 5:**
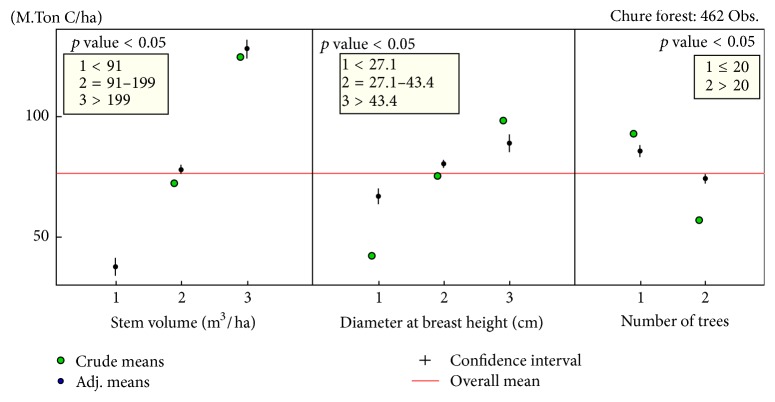
The confidence interval plots of the variables: stem volume, dbh, and the tree frequency.

A 95% confidence interval plot of three statistically associated determinants, stem volume, dbh, and the number of trees, is shown in Figure 5. The red horizontal line is the overall mean carbon stock. Confidence intervals of each variable from the model are shown in black vertical lines with the black dots that represent the adjusted means of the variables. The green dots are the crude means of the variables. The stem with its volume ≥200 m^3^/ha had the highest carbon stock which gradually decreased as the stem volume was reduced. Similarly, the dbh/carbon content association inferred that more than 43.4 cm had highest amount of carbon in Chure forest. It showed similar results to number of trees present in the subplots. When the number was more than 20, the carbon stock tends to remain higher than when the number was below that.

**Figure 6:**
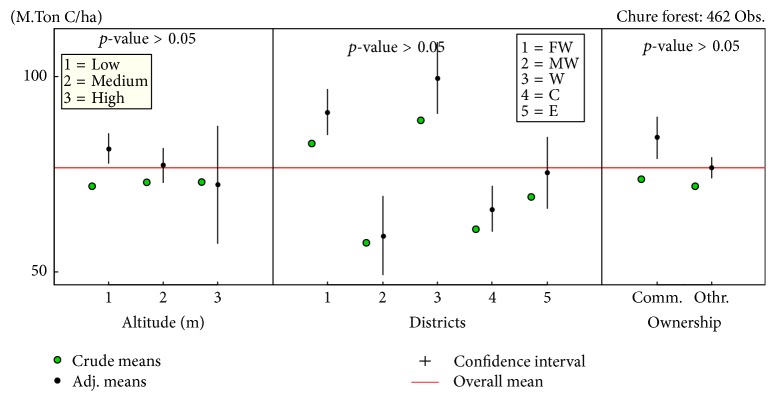
Confidence interval plots of the variables: stem altitude, district, and ownership of the forest.

However, the variables altitude, districts, and ownership of the forest did not show any significant association with carbon stock of the forest (Figure 6).

## 4. Discussions

The Ministry of Forest and Soil Conservation, government of Nepal, declared that the stock is 372 tC/ha in the country [28]. The International Panel for mitigating climate change reports that the carbon stock, in Nepal, is 369 tC/ha [29]. The Department of Forest Research and Survey, the government of Nepal, reports that Chure has 116.94 tC/ha carbon stock [30]. But, in this study, the carbon stock of Chure is 76 tC/ha. This difference was found since it considered only the stem volume with diameter more than 5 cm. In addition, carbon stock per unit area can vary depending on several factors such as geographic location [3], density of trees [2], species diversity [11], stem volume in forest, dbh [7, 31], canopy [10], and other managerial or legal aspects [12] of the forest.

In this study, out of six predictors, the stem volume, more than 199 m^3^/ha, was associated with the highest carbon content than the other categories. A study in Mexico has found a positive correlation between basal area and the carbon stock [7]. Stem is the main part where majority of tree carbon gets stocked; therefore, the volume of stem matters for stocking carbon in a forest. The trees having dbh more than 43.4 cm had highest carbon stock and those having less than 27.1 cm showed lowest carbon content. A study in Southern China revealed that medium to large diameter class trees contributed predominantly to biomass carbon accumulation while the others, such as seedlings and saplings, contributed very small proportion [31]. Brown has statistically proved that diameter at breast height (dbh) alone explains more than 95% of the variation in aboveground tropical forest carbon stocks, even in highly diverse regions [3]. Besides, the government of Nepal has declared Chure region as an Environmental Conservation Area and hence the deforestation might have reduced in this forest.

The number of trees per plot has significant association with the total carbon content. Less than 20 trees per plot showed higher carbon stock and more than that showed lower carbon content in the forest. The result is consistent with a study in China that explained the fact that very lower number of trees (with medium and higher stem diameter) in forest possessed higher carbon stock than in the crowded forest containing more tree saplings and seedlings [31]. While the study around Servarayan hills of India contrasted with our results that the number of trees positively correlated with the carbon content in forests [8]. This difference might be due to the heterogeneity of type of forest and the characteristics of the species.

No statistical significance was seen between carbon stock and the altitude of the forest area. The graph shows that lower the altitude is the higher the carbon stock in forest is. This may be due to comparatively more clay rich soil towards the southern low altitude belt of Chure. There is homogeneity in distribution of altitude and vegetation throughout east to west of the Chure belt. The northern belt is uniformly higher than the southern one and the vegetation type is accordingly distributed. In contrast, the studies in India and Tanzania had found that the carbon stock significantly increased by the altitude of the forest area [8, 9]. However, in both literatures, composition variations of tree species were found with the altitude.

This study explored that there was no statistically significant variation of carbon stock among districts. A study in India found that the carbon stock was significantly associated with the variation in location, meaning that the carbon stock was found higher in northern than in the southern aspects [2], which were completely different climate zones. However, in Chure, since the climate zone is homogeneous throughout the belt from east to west, that makes no basic difference in the characteristics of tree wood and associated carbon stock.

The ownership of the forest and the carbon content in the Chure forest showed no statistical correlation. The carbon stock did not differ significantly in community owned or the other type of forests. The reason might be that the whole Chure belt forest, regardless of its ownership type, is an environmentally protected area abided by Environment Protection Act 2015 of Nepal government. So, the Chure showed no difference in quality and quantity of vegetation. This result was found contrasting with the study on 80 forest commons in 10 countries across Asia, Africa, and Latin America, which had found a significant association between livelihood benefits and carbon storage. The autonomous forestry (community owned) had higher carbon stock than any other type in all three continents [12]. This study considered only the stem volume to find the carbon content, and that might slightly reduce the net carbon stock (cumulative one of stem, branches, and leaves) of the forest. Topographic factor is important in this issue; however, the soil, an important factor, in Chure is very dry and mostly contains sandy loam. Since the rate of water infiltration is very high minimum amount of clay is left in it. This has reduced the possibility of being a good source of soil carbon stock in Chure range. This work limits the effect of other topographic factors in calculating the forest carbon stock, which is planned to continue in the next step.

## 5. Conclusions

The carbon stock in Chure belt has shown significant statistical association with the factors like dbh, stem volume, and the frequency of trees in the forest. The higher the sv and dbh in forest wood, the higher the carbon stock in that forest, while more number of trees indicated the lower carbon stock. Since carbon stock showed no statistical association with altitude, districts, and the ownership, the carbon in the forest can be enhanced by either increasing sv and dbh of the wood or reducing the tree density within the forest. This would be beneficial for REDD+ program and supports the national environmental policy of reducing deforestation and forest degradation. Chure is fragile and vulnerable to erosions. However, this belt has a natural protective role to control erosion of a lot of natural resources like soil, sand, and gravel. The enhancement of carbon stock through forest conservation not only is a way to restore a lot more natural resources indirectly in this area but also can provide a clear picture of how forest carbon can be increased by manipulating the associated variables and that can support UN-REDD+ program as well. The results would be valuable for government, regarding carbon conservation issues around Chure forest.

## Figures and Tables

**Table 1:** Outline of floristic parameters in study area.

Floristic parameters	Existing status
Total tree species	281 species in study area
Number of stems having dbh ≥5 cm	731/ha
Average stem volume	153.99 m^3^/ha
Live trees, aboveground air dried biomass	179 t/ha
Total forest clusters	121
Total subplots	476
Total area of each subplot (*r* = 20 m)	1256.6 m^2^
Average number of trees per subplot	20
Total community forest subplots	249

**Table 2:** Descriptive statistics of carbon, stem volume, dbh, altitude, and tree frequency.

Factors	Min.	Max.	Median	Mean	Standard deviation
Carbon (M.ton/ha)	0.63	264.6	72.68	76.67	44.58
Stem vol. (m^3^/ha)	1.14	509.7	147.6	154.40	86.45
dbh (cm/plot)	5.4	109.2	35.1	36.35	14.24
Altitude (m/plot)	124	1632	479	513.3	289.47
Tree freq.	1	52	19	19.48	8.82

**Table 3:** Detailed result of variables after multiple regression model.

Variables (category)	Coefficients	Standard error	*p* value
Stem volume (3)			
SVol-1	1		
SVol-2	37.0299	2.1099	<0.05
SVol-3	82.7046	2.9667	<0.05
Stem diameter (3)			
Dbh-1	1		
Dbh-2	12.6649	1.9182	<0.05
Dbh-3	19.8899	2.6723	<0.05
Number of trees (2)			
Noftr-1	1		
Noftr-2	−10.7735	1.8959	<0.05
Altitude (3)			
Alt-1	1		
Alt-2	3.1432	1.5511	<0.05
Alt-3	−3.5070	3.2964	0.2880
District (5)			
Dist1	1		
Dist2	−1.2887	2.5924	0.6193
Dist3	1.2523	2.4051	0.6028
Dist4	1.1656	1.9662	0.5536
Dist5	4.0286	2.4128	0.0957
Ownership (2)			
Ownsp-1	1		
Ownsp-2	−0.6158	1.6022	0.7009
